# Supplementary material for: Sialic Acid and Colchicine Functionalized Silica Nanoparticles: A Novel Approach to Leishmanicidal Selective Treatments
Source: Biomedicines. 2025 Jul 6;13(7):1648. doi: 10.3390/biomedicines13071648 (PMC12292207; doi:10.3390/biomedicines13071648)
Supplement: Supplementary file 1 [file biomedicines-13-01648-s001.zip › biomedicines-3692964-supplementary.pdf]

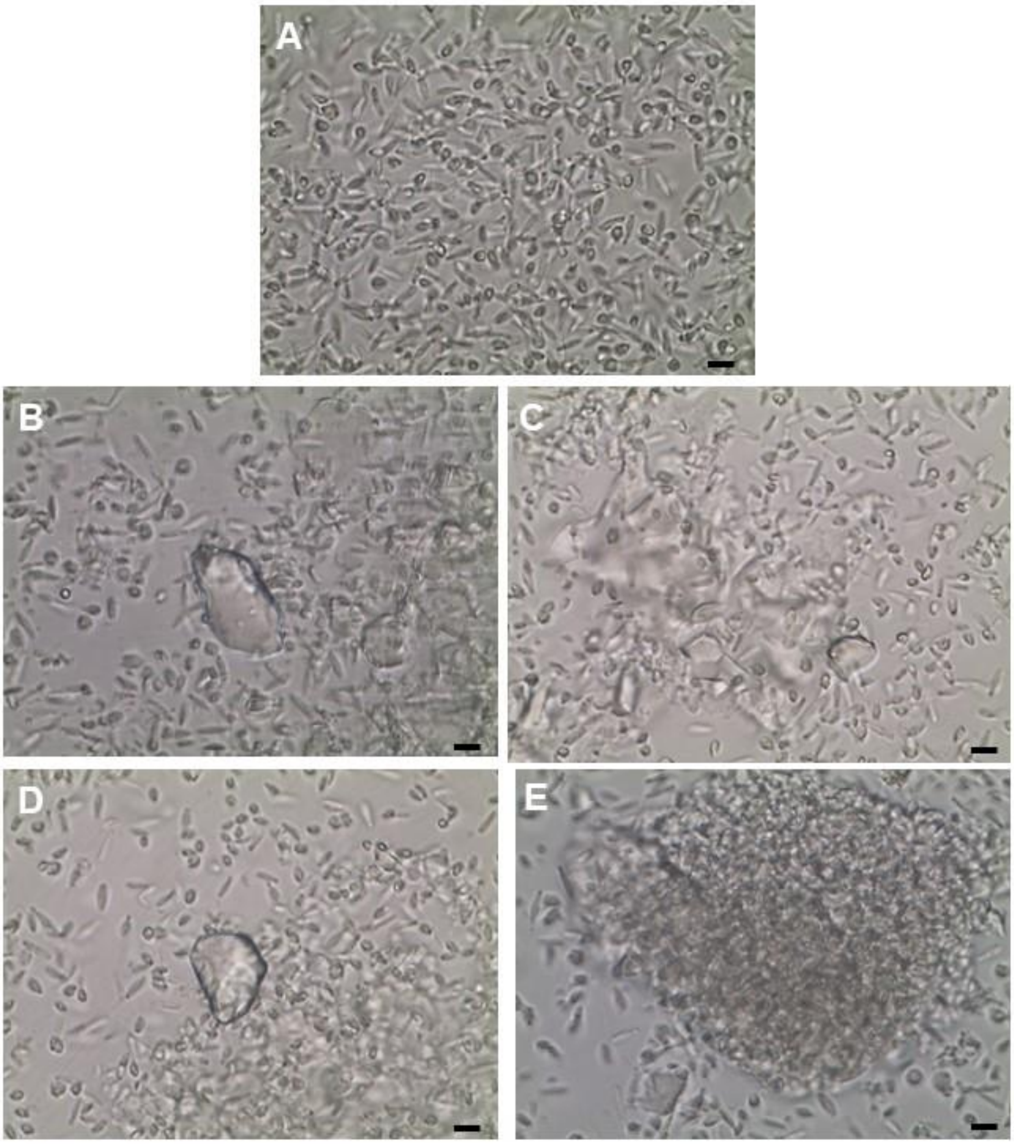


**Figure S1.** Leishmania braziliensis promastigote-nanoparticle binding by light microscopy. (**A**) Control of *L. braziliensis* promastigote in vitro culture, (**B**,**C**) promastigote of *L. braziliensis* promastigote treated with MSN-AS at 0.1 mg/mL, (**D**) promastigote of *L. braziliensis* promastigote treated with MSN-AS-COL at 0.1 mg/mL and (**E**) promastigote of *L. braziliensis* promastigote treated with MSN-AS-COL at 0.5 mg/mL Bar: 10 μm.
